# Studying the Interaction between Bendamustine and DNA Molecule with SERS Based on AuNPs/ZnCl_2_/NpAA Solid-State Substrate

**DOI:** 10.3390/ijms241713517

**Published:** 2023-08-31

**Authors:** Lina Yao, Yanjie Li, Zhenzhong Zuo, Ziyi Gong, Jie Zhu, Xiaoqiang Feng, Dan Sun, Kaige Wang

**Affiliations:** State Key Laboratory of Cultivation Base for Photoelectric Technology and Functional Materials, National Center for International Research of Photoelectric Technology & Nano-Functional Materials and Application, Key Laboratory of Photoelectronic Technology of Shaanxi Province, Institute of Photonics and Photon-Technology, Northwest University, Xi’an 710127, Chinasund@nwu.edu.cn (D.S.)

**Keywords:** surface-enhanced Raman spectroscopy, solid-state substrate, bendamustine, calf thymus DNA, drug–DNA interaction

## Abstract

Bendamustine (BENDA) is a bifunctional alkylating agent with alkylating and purinergic antitumor activity, which exerts its anticancer effects by direct binding to DNA, but the detailed mechanism of BENDA–DNA interaction is poorly understood. In this paper, the interaction properties of the anticancer drug BENDA with calf thymus DNA (ctDNA) were systematically investigated based on surface-enhanced Raman spectroscopy (SERS) technique mainly using a novel homemade AuNPs/ZnCl_2_/NpAA (NpAA: nano porous anodic alumina) solid-state substrate and combined with ultraviolet–visible spectroscopy and molecular docking simulation to reveal the mechanism of their interactions. We experimentally compared and studied the SERS spectra of ctDNA, BENDA, and BENDA–ctDNA complexes with different molar concentrations (1:1, 2:1, 3:1), and summarized their important characteristic peak positions, their peak position differences, and hyperchromic/hypochromic effects. The results showed that the binding modes include covalent binding and hydrogen bonding, and the binding site of BENDA to DNA molecules is mainly the N7 atom of G base. The results of this study help to understand and elucidate the mechanism of BENDA at the single-molecule level, and provide guidance for the further development of effective new drugs with low toxicity and side effects.

## 1. Introduction

**Figure 1:**
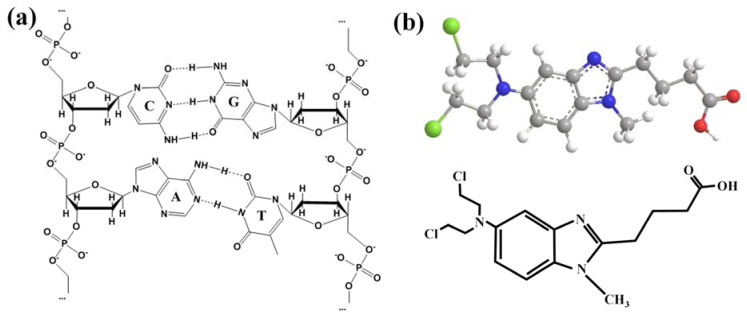
(**a**) Chemical structure diagram of DNA; (**b**) Schematic diagram of the structure of BENDA.

Deoxyribonucleic acid (DNA) is the coding molecule used to store, transmit, and express genetic information and is one of the main targets of drug therapy [1]. As shown in Figure 1a, DNA strands are composed of several deoxyribonucleotides polymerized by 3′→5′ phosphodiester bonds, with a basic backbone formed by alternating deoxyribose and phosphate connections on the outer side and four kinds of bases (adenine, guanine, cytosine, and thymine) on the inner side. The bases between the two chains are connected by hydrogen bonds to form base pairs [2]. Drug molecules can affect or damage the structure of DNA through different pathways, which in turn directly or indirectly impede the normal DNA replication, transcription, and other processes, and eventually cause apoptosis to exert anti-tumor effects [3,4]. It has been shown that drugs that interact with DNA in vivo can also act in vitro [5]. Therefore, the interaction between DNA and drugs can be studied in vitro to predict the mechanism of action of both occurring in vivo. At this stage of research, researchers prefer to use calf thymus DNA rather than human DNA for their experiments because ctDNA is a natural, high-quality dsDNA template from a mammalian source that is widely available, easy, and inexpensive to source [6] and thus ctDNA is widely used for drug–DNA interaction spectroscopy, polymerase analysis, and other biomolecular experiments [7]. Therefore, in order to better explore the correlation between anticancer activity and structure, it is particularly important to understand the interaction properties of DNA and ligands [8], At the same time, it provides a new idea for designing a hybrid nanomaterials drug delivery system based on DNA structure [9].

Bendamustine (BENDA), a bifunctional alkylating agent with anticancer efficacy, was synthesized in Germany in early 1963. It was widely used for the treatment of lymphoma and myeloma in Eastern Europe from 1970 to 1980, but was not studied in clinical trials until 2000 [10]. In 2008, bendamustine was approved by the US Food and Drug Administration for the treatment of chronic lymphocytic leukemia (CLL) and inert B-cell non-Hodgkin’s lymphoma (NHL), facilitating its development in the field of cancer therapy [11]. BENDA is composed of three elements: a 2-chloroethylamine alkylation group, a benzimidazole ring, and a butyric acid side chain (Figure 1b), and stops the growth of cancer cells by binding to DNA and interfering with its normal activity. The unique structure makes BENDA more stable than other nitrogen mustard drugs, which can cause more lasting DNA damage. This structure may be directly related to its specific anticancer activity and clinical differences [12].

The medicinal mechanism of BENDA has been studied by researchers for decades: in 1996, Strumberg et al. [13] tested in vitro DNA double-strand breaks in seven cancer cells using pulsed-field gel electrophoresis with BENDA, cyclophosphamide, and melphalan. The results showed that BENDA caused a higher number of DNA breaks and better inhibition of subsequent DNA repair mechanisms at the same half-inhibitory concentration setting. In 2001, Barman Balfour et al. speculated that the benzimidazole ring in the structure of BENDA could provide a specific contribution in its medicinal use and suggested that this could be one of the reasons for its unique anticancer efficacy [14]. In 2004, Leoni et al. [15] analyzed the effect of BENDA on the gene expression profile of SU-DHL-1 cells, a type of lymphoma cell, after its action on them, and showed that BENDA induces gene expression for intracellular related activities, such as DNA replication and life cycle. Then, in 2008, they made a correlation calculation between BENDA and 25 other alkylating agents by using the novel algorithm of Compare Analysis [16]. The correlation coefficients indicated that BENDA has a potentially unique mechanism of action compared to most conventional alkylating agents, which could help explain the lack of complete cross-resistance between it and other alkylating agents.

To investigate this uniqueness, Leoni et al. framed the series of changes induced by BENDA in NHL cancer cells (e.g., apoptotic pathways, DNA damage repair mechanisms, etc.). The results showed that BENDA not only caused strong activation of the p53 gene (an oncogene in humans) in cells, but also initiated two pathways, inhibition of mitotic checkpoint and induction of apoptosis. Furthermore, unlike the DNA repair by alkyltransferases activated by conventional alkylating agents, BENDA activates base–excision DNA repair and is based on a complex cellular response mechanism with a high probability that resistance does not readily emerge. In 2011, Leoni and Hartley et al. [17] summarized the understanding and hypotheses regarding the mechanism of action of BENDA, and although these studies provided partial explanation for the mechanism, more experiments are still needed to investigate such important questions as the damage caused by BENDA to DNA. In the same year, Leoni and Cheson [18] confirmed the hypothesis that the benzimidazole ring is a structural component unique to BENDA itself and showed that the introduction of a heterocyclic structure allows BENDA to better seek out and penetrate DNA structures for prolonged retention, which is relevant to its anticancer activity.

Drug–DNA binding studies have been described in several papers and there are several research methods, including atomic force microscopy (AFM), ultraviolet–visible spectrometry (UV–vis), fluorescence spectroscopy, and isothermal titration calorimetry (ITC) [19,20,21,22], which can obtain information about the binding mode, general image, or conformational changes of drug–DNA interactions, while detection at the single-molecule level has certain limitations. Compared to the previous several methods, Raman spectroscopy has received wide attention for its advantages of narrow spectral identification and providing molecular structure information. Moreover, the discovery of surface-enhanced Raman spectroscopy (SERS) can further obtain the primary/secondary structures present in the molecules of interest, which greatly improves the detection sensitivity of Raman spectroscopy, even to the level of single-molecule detection [23]. In 2016, Hoda Ilkhani et al. [24] studied the interaction of doxorubicin (DOX) with double-stranded DNA of breast cancer using SERS, and the spectral changes reflected the formation of DOX embedded in double-stranded DNA and DNA-DOX complexes, and it was found that in the occurrence of chimeric binding, the loops B and C of DOX were inserted between guanine and adenine bases, while ring A is outside the duplex and the glycosyl group stays in the small groove and interacts with the base pair near the center of the intercalation. In 2015, Shweta Agarwal et al. [25] reported SERS bands associated with DNA complexation of chloroethyl nitrosourea derivative (nimustine and semustine) using SERS as an analytical tool, revealing that both drugs are associated with double-stranded DNA by heterocyclic nitrogenous base pairs and also indicated the formation of dG–dC interstrand crosslinks in the DNA double helix structure.

In this study, calf thymus DNA (ctDNA) and its interaction with the anticancer drug BENDA were investigated using a novel AuNPs/ZnCl_2_/NpAA (NpAA: nano porous anodic alumina) solid substrate. First, we prepared NpAA substrates by a standard two-step anodic oxidation method and grew ZnCl_2_ thin film layers and gold nanoparticles (AuNPs) sequentially on them, and then morphologically and electrically characterized the prepared substrates and tested some indicators of the substrates using rhodamine 6G (R6G) as a probe molecule to demonstrate that the substrates possess single-molecule detection levels. Finally, the substrates were applied to the SERS spectroscopy study of BENDA binding to DNA, and different drug/DNA molar ratios were designed in the experiments to determine, among others, the site of action and structural effects of BENDA on DNA. The peak changes indicated a groove binding mode of interaction between BENDA and DNA, with the N7 atom in the G base as the main site of binding for both. The binding model of BENDA–DNA interaction can be derived in combination with molecular docking studies, and this study may support rational drug derivative design.

## 2. Results and Discussion

### 2.1. AuNPs/ZnCl_2_/NpAA Solid-State Substrate

#### 2.1.1. Substrate Preparation

**Figure 2:**
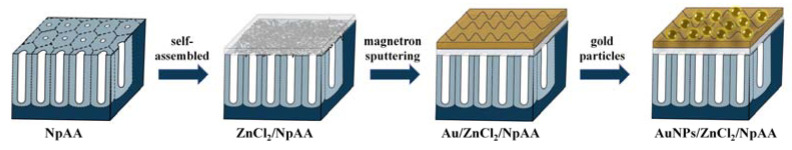
AuNPs/ZnCl_2_/NpAA schematic diagram of substrate preparation process.

As shown in Figure 2, the preparation steps of the AuNPs/ZnCl_2_/NpAA solid-state substrate in experiment were divided into four parts:

1. The NpAA substrate was prepared by the standard two-step anodic oxidation method [26,27]. Add electrolyte into the self-made glass tank and put a magnet for magnetic stirring, place the glass tank in the low-temperature cooling tank, connect the external 40 V voltage regulator power supply, set the temperature of the low-temperature tank to 0 °C during anodic oxidation, the electrolyte added in the glass tank is 0.3 mol/L oxalic acid solution, the first oxidation time is 1 h, the second oxidation time is adjusted to 2 h;
2. ZnCl_2_ layers were prepared on the surface of NpAA by self-assembly method. First, ZnCl_2_ solution with a concentration of 0.05 mol/L was prepared, and then the ZnCl_2_ solution was added to the NpAA substrate surface by drops until it was completely covered. Finally, the whole substrate, after being added with ZnCl_2_ solution, was placed in a closed room temperature environment for 7 days, and the surface would naturally form nanosheet structures;
3. A layer of Au film of about 30 nm was plated on the surface of the substrate after the previous step by magnetron sputtering;
4. Gold particles AuNPs with a particle size of 50 nm were used for modification on the uppermost layer of the substrate, which was first soaked in 2% PVP ethanol solution for 6 h, rinsed, and then transferred to AuNPs solution for 2 times, both for 6 h.

#### 2.1.2. Substrate Characterization

**Figure 3:**
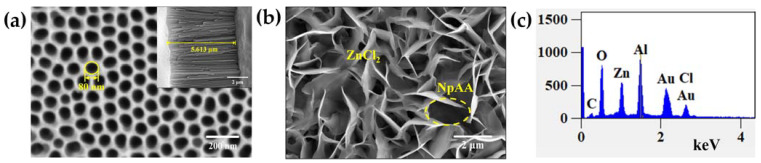
(**a**) Surface SEM morphology of NpAA, the illustration shows the cross-sectional shape; (**b**) SEM morphology of the surface of the ZnCl_2_/NpAA substrate, the yellow circle represents NpAA; (**c**) Energy dispersive spectrogram of AuNPs/ ZnCl_2_/NpAA solid substrates.

The surface morphologies of the prepared NpAA and ZnCl_2_/NpAA were characterized by scanning electron microscopy (SEM), as shown in Figure 3a,b, respectively. As can be seen from Figure 3a, there are tightly connected, ordered, and uniform primitive nanopore structures on the substrate, which are closely aligned with hexagonal cells. The pore size is relatively uniform, maintaining between 80 and 90 nm. The illustration at the top right shows a cross-section of the substrate, with the nanopore channels at the bottom of the vertical clearly visible, with channel lengths remaining between 5.5 and 6 microns. In the preparation process, the aperture can be controlled by changing the type of electrolyte and the reaction time.

After the self-assembly of ZnCl_2_ solution on the NpAA surface is completed (Figure 3b), a uniform three-dimensional intersecting nanosheet structure will be formed on the surface, covering all the pore structures and forming a “hot spot” region. The elemental species analysis of the tiny regions of the AuNPs/ZnCl_2_/NpAA substrate after AuNPs modification was performed by the self-contained energy spectrometer in SEM. Figure 3c shows the elemental energy spectrum of the AuNPs/ZnCl_2_/NpAA substrate, which indicates that the substrate contains five elements: Al, O, Zn, Cl, and Au. Of these, Au is derived from a gold film plated on the surface of the substrate, Al and O are both derived from the underlying NpAA, and Zn and Cl are attributed to the stereocrossing ZnCl_2_ nanosheets.

#### 2.1.3. Substrate Performance

To test the performance of the active substrate of AuNPs/ ZnCl_2_/NpAA, we used R6G as the test substance to examine the sensitivity and Enhancement Factor (EF) of this substrate [28]. R6G is one of the most commonly used probe molecules to detect the performance of SERS substrates with high adsorption properties and distinct Raman peaks.

**Figure 4:**
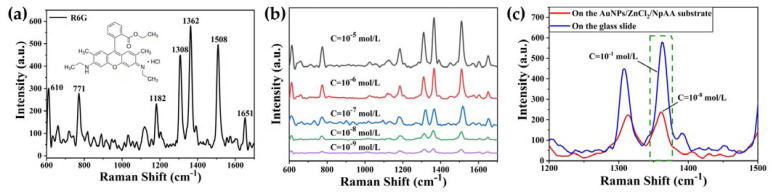
Raman spectra of R6G solutions on different substrates: (**a**) quartz substrate with R6G concentration of 10^−1^ mol/L, inset shows the chemical structure of R6G; (**b**) AuNPs/ZnCl_2_/NpAA substrate with R6G concentration of 10^−6^ mol/L-10^−9^ mol/L; (**c**) local magnification of both substrates (1200 cm^−1^–1500 cm^−1^), the green box shows the selected peak at 1362 cm^−1^.

Figure 4a shows the Raman spectrum of the R6G solution (10^−1^ mol/L) on a quartz substrate, and it is evident that most of its Raman characteristic peaks are located between 600 cm^−1^ and 1700 cm^−1^, mainly 610, 771, 1182, 1308, 1362, 1508, and 1651 cm^−1^, all of which characterize part of the structural information of R6G [29,30]. Among them, the spectral peaks at different positions are attributed as follows: 610 cm^−1^ belongs to C-C-C ring bending vibrations, 771 cm^−1^ and 1182 cm^−1^ characterize C-H out-of-plane and in-plane bending vibrations, respectively, 1308 cm^−1^ is attributed to N-H in-plane bending vibrations, 1362 cm^−1^ represents C-N stretching vibrations, and 1508 cm^−1^ and 1651 cm^−1^ both belong to the aromatic C-C stretching vibrational state. Figure 4b shows the SERS spectra of the R6G solution with the concentration decreased from 10^−5^ mol/L to 10^−9^ mol/L. At this time, R6G was located on the AuNPs/ZnCl_2_/NpAA substrate, and the main characteristic peaks were still clearly visible at the low concentration (10^−9^ mol/L), which indicated that the detection limit of this substrate for R6G was up to 10^−9^ mol/L.

As shown in Figure 4c, the strong Raman peak at 1362 cm^−1^ in the R6G solution placed on a glass slide (concentration of 10^−1^ mol/L, blue spectral line) and on the AuNPs/ZnCl_2_/NpAA substrate (10^−9^ mol/L, red spectral line) was selected as the characterization peak for the calculation of EF, respectively. Keeping the same detection conditions (e.g., exposure time, laser power, etc.) in the experiment, the EF of the SERS substrate can be calculated by the following standard equation:

(1) $$EF=\frac{I_{SERS}C_{Raman}}{I_{Raman}C_{SERS}}$$

where $I_{SERS}$ and $I_{Raman}$ correspond to the peak intensities in the SERS spectra and ordinary Raman spectra of R6G solution, respectively, and $C_{SERS}$ and $C_{Raman}$ correspond to the concentrations of R6G solution in the SERS analysis and ordinary Raman analysis, respectively. From Figure 4c, $I_{SERS}$ and $I_{Raman}$ are 238 and 579, respectively, which can be substituted into Equation (1) to obtain the EF of AuNPs/ZnCl_2_/NpAA substrate of about 4.11 × 10^7^, which indicates that this SERS substrate has a strong enhancement effect.

### 2.2. SERS Spectrum

#### 2.2.1. SERS Spectrum of ctDNA

**Figure 5:**
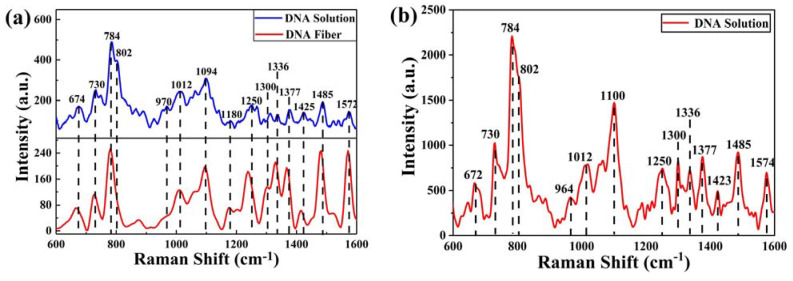
(**a**) Raman spectra of ctDNA in both fiber and solution (concentration of 9.5 × 10^−2^ mol/L); (**b**) SERS spectra of ctDNA when diluted to a concentration of 10^−6^ mol/L.

Figure 5a shows the Raman spectra of ctDNA in the states of fiber and solution (concentration of 9.5 × 10^−2^ mol/L), and it can be seen that the main Raman peaks of ctDNA solution contain 674, 730, 784, 802, 970, 1012, 1064, 1094, 1180, 1250, 1300, 1336, 1377 1425, 1485, and 1572 cm^−1^, all of which belong to the relevant characterization of the molecular vibrational information in part of the ctDNA structure [31,32,33]. Among them, the spectral peaks at different positions are attributed as follows: 674, 730, 1180, 1250, 1300, 1336, 1377, 1425, 1485, and 1572 cm^−1^ are attributed to base-related vibrations; 784, 802, and 1094 cm^−1^ characterize phosphate group-related vibrations; 1012 cm^−1^ and 1064 cm^−1^ are caused by C-O stretching vibrations of deoxyribose; 674 cm^−1^ and 730 cm^−1^ correspond to symmetric vibrations of bases T and A, respectively; and 1180 cm^−1^ is attributed to C-N bond stretching outside the bases. In addition, 1250 cm^−1^ and 1300 cm^−1^ characterize the vibrational modes of bases A and C and bases C and G, respectively; 1336 cm^−1^ is attributed to the molecular vibration of base A; and 1377 cm^−1^ corresponds to the molecular vibration of the three bases T, A, and G. 784, 802, and 1094 cm^−1^ indicate the O-P-O chemical bond in the phosphate group, the main chain, and PO_2_, respectively, of the O=P=O double bond, and 1012 cm^−1^ belongs to the C-O single bond stretching vibration in deoxyribose. After the ctDNA fiber was prepared into solution, the 970 cm^−1^ peak characterizing deoxyribose also appeared in the SERS spectrum, except that most of the peaks were still present, and some peaks were slightly shifted.

The SERS spectra of ctDNA solution diluted to 10^−6^ mol/L are shown in Figure 5b. Comparing with the spectra before dilution, it can be seen that the SERS spectra retain most of the original peaks after the active substrate enhancement, despite the low concentration, and provide higher peak intensities while eliminating most of the interference caused by the non-characteristic peak noise. The peaks at 970, 1094, and 1305 cm^−1^ were changed to 964, 1100, and 1300 cm^−1^, respectively, and the peak at 1180 cm^−1^ disappeared. This may be attributed to the C-N bond adsorbed on the surface of SERS substrate (whose vibration mode was parallel to the base), resulting in the disappearance of the strip [34]. This indicates that the base portion of the ctDNA structure is closer to the substrate surface, and that it is mainly adsorbed to the SERS substrate surface as the base portion.

#### 2.2.2. SERS Spectrum of BENDA

**Figure 6:**
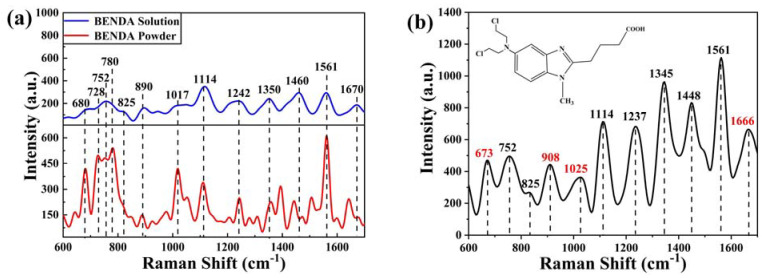
(**a**) Raman spectra of BENDA in both powder and solution (concentration of 5 × 10^−2^ mol/L); (**b**) SERS spectra of diluted BENDA solution at a concentration of 10^−6^ mol/L. The inset shows the chemical structure of BENDA.

Figure 6a shows the Raman spectra of BENDA powder and solution (concentration 5 × 10^−2^ mol/L). It can be seen that the main Raman peaks of BENDA solution are concentrated in the range of 600 cm^−1^ to 1700 cm^−1^, which mainly contain the following peaks: 680, 728, 752, 780, 825, 890, 1017, 1114, 1242, 1350, 1460, 1561, and 1670 cm^−1^.

Based on the molecular structure composition of BENDA, it is known that the spectral peaks at different positions are attributed as follows [35]: 680 cm^−1^ is attributed to C-Cl bond stretching vibration, 728 cm^−1^ characterizes the C-C stretching vibrational state, 752 cm^−1^ is attributed to CH_2_ group, 780 cm^−1^ belongs to CH_2_ group swinging vibration mode, 825 cm^−1^ and 890 cm^−1^ are both attributed to C-H chemical bond, the former in the rocking vibration mode and the latter in the out-of-plane bending vibration mode. In addition, 1017 cm^−1^ is attributed to the CH_2_ group, 1114 cm^−1^ is attributed to the benzene ring breathing, 1242 cm^−1^ characterizes the benzene ring vibration, 1350 cm^−1^ is attributed to the C-N stretching and C-H deformation vibration, and 1460 cm^−1^ is attributed to the antisymmetric bending vibration of the CH_3_ group. Further, 1561 cm^−1^ is a common feature of C-C stretching and H-C-H vibrations, and 1670 cm^−1^ is the stretching vibrational state of C=C and C=N double bonds.

Figure 6b shows the SERS spectra of BENDA solution after being diluted to 10^−6^ mol/L. The inset shows the chemical molecular structure of BENDA. Comparing the spectra before dilution, it can be seen that the peaks of 680 cm^−1^ and 1670 cm^−1^ become 673 cm^−1^ and 1666 cm^−1^, respectively, and two new peaks of 908 cm^−1^ (C-C) and 1025 cm^−1^ (pyridine ring respiration vibration) appear after the application of active substrate enhancement.

#### 2.2.3. SERS Spectrum of BENDA-ctDNA

**Figure 7:**
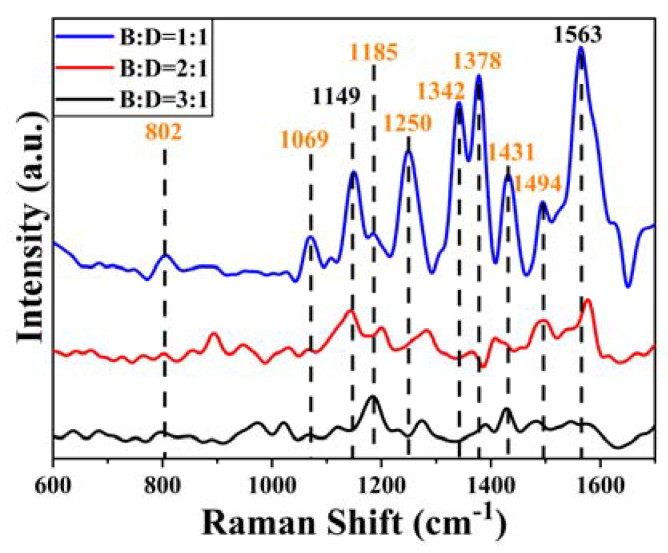
SERS spectra of BENDA−ctDNA complexes at different molar ratios (1:1, 2:1, 3:1).

Figure 7 shows the SERS spectra of the mixed solutions of BENDA and ctDNA at different molar ratios (1:1, 2:1, 3:1), and it can be seen that the main characteristic peaks of the BENDA-ctDNA complex contain 802, 1069, 1149, 1185, 1250, 1342, 1378, 1431, 1494, and 1563 cm^−1^, and the peak attribution of each characteristic peak is listed in Table 1.

A comparative analysis of the SERS spectra of ctDNA (Figure 5b), BENDA (Figure 6b), and the BENDA–ctDNA complex (Figure 7) shows that the peaks at 802, 1069, 1185, 1250, 1342, 1378, 1431, and 1494 cm^−1^ are attributed to ctDNA (orange numbers in Figure 7), while the characteristic peaks at 1149 cm^−1^ and 1563 cm^−1^ are attributed to BENDA. The absence of the highest peak in the DNA at 784 cm^−1^ indicates that the interaction between BENDA and the phosphate groups in ctDNA may have been disrupted or altered, so the phosphate clamp mechanism is possible [36]. In the BENDA–ctDNA complex, the Raman peak at 802 cm^−1^ was not shifted at low BENDA concentrations, while at higher concentrations, a frequencies shift of ~4 cm^−1^ occurred in the spectral band, and there was a tendency for a significant decrease in the peak intensity, indicating a partial perturbation effect of BENDA on the phosphate backbone of the DNA structure, especially at its higher concentration. In addition, the Raman peak at 1069 cm^−1^ was only slightly shifted (about ±2 cm^−1^), but a significant decrease in peak intensity occurred. Combined with the peak attribution at 1069 cm^−1^, it is clear that the binding changes of the deoxyribose–phosphate backbone all indicate some external binding of BENDA to the DNA double helix structure.

The Raman band observed at 1494 cm^−1^ is mainly attributed to the N7 atom of the G base in DNA, which is a major groove-labeled band [37] and is sensitive to interactions in which groove binding is the main binding mode, and the intensity of this peak gradually decreases and redshifts to 1485 cm^−1^ when the molar concentration of BENDA increases, indicating that the hydrogen bonding binding environment in the G base is changed. The intensity of the characteristic peaks at 1185 cm^−1^ (extra-base C-N stretch) and 1431 cm^−1^ (A) [38] both show a decrease and then an increase at progressively increasing concentrations of BENDA, the decrease may be due to the aggregation and condensation of DNA at higher concentrations of BENDA, which increases the interstrand attraction and leads to a decrease in the polarization rate of the DNA bases and their externals. Later, higher concentrations of BENDA lead to destabilization of the DNA double helix state, which in turn leads to a decrease in the interstrand attraction and eventually to an increase in the polarization rate of the bases and their exterior.

### 2.3. UV–Vis Absorption Spectroscopy

- ctDNA concentration

**Figure 8:**
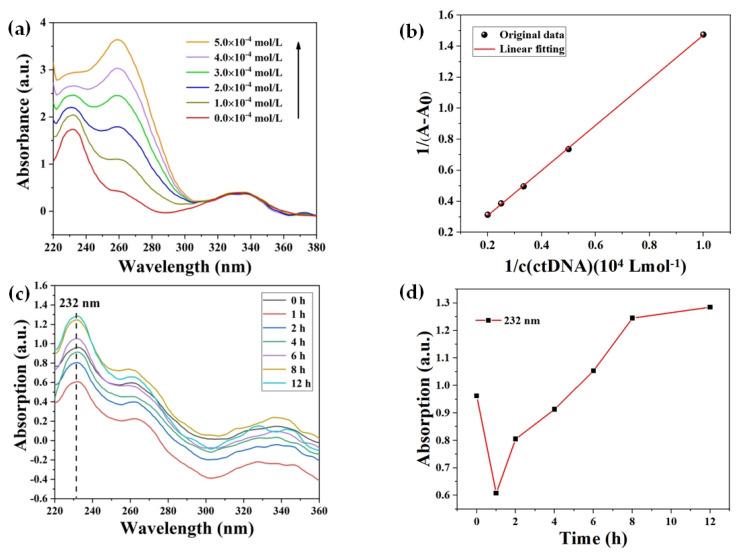
(**a**) UV–vis absorption spectra of BENDA–ctDNA complexes measured at different ctDNA concentrations; (**b**) linear variation of $1/(A-A_{0})$ with 1/[DNA]; (**c**) UV–vis absorption spectra of BENDA–ctDNA complexes at different times; (**d**) absorbance of BENDA–ctDNA complexes at 232 nm at different times.

As shown in the spectrum of the red line in Figure 8a, the UV–vis absorption peaks of BENDA were distributed at 232 nm, 262 nm, and 336 nm, with the strongest absorption peak at 232 nm. With the increasing concentration of ctDNA in the mixed system, the wave peaks at 232 nm and 262 nm showed a hyperchromic effect and a blue shift of about 2 nm at 262 nm. Normally, the absorption spectra of small molecules did not change after the addition of DNA solution to the mixed solution, which showed that the main binding mode of small molecules and DNA was electrostatic binding. If the spectra of the mixed solution showed a significant change after the addition of DNA solution, it indicates that the main way of their binding is intercalative binding or groove binding. When the hypochromic effect and the red shift phenomenon (≥10 nm) appear simultaneously, it indicates that the two are mainly combined in the way of intercalation, otherwise, they are groove binding [39]. In the BENDA-ctDNA mixed system solution, only the spectrum at 232 nm showed a significant change without a significant redshift. Only when both the hypochromic or hyperchromic effect and the obvious red shift appeared simultaneously, we considered the drug molecule and ctDNA as intercalative binding. From this, we can conclude that the interaction mechanism between BENDA and ctDNA is mainly groove binding in our series of experiments [40].

To have a further understanding of the binding strength of BENDA and ctDNA [41], we obtained Figure 8b based on the UV–vis spectral data at 232 nm in Figure 8a by a linear fit to Equation (2):

(2) $$\frac{1}{A-A_{0}}=\frac{1}{A}+\frac{1}{K_{A}\times A\times \left[DNA\right]}$$

where $A_{0}$ corresponds to the absorbance of free BENDA at 232 nm, A is the absorbance of free BENDA at 232 nm in the presence of different concentrations of ctDNA, [DNA] refers to different concentrations of ctDNA, and the ratio of the slope $1/\left(K_{A}\times A\right)$ to the intercept 1/A can be used to estimate the bonding constant $K_{A}$ for the interaction of BENDA with ctDNA, which was calculated to $K_{A}$ ≈ 1.23 × 10^3^ L/mol (R^2^ = 0.98).

- Reaction time

Figure 8c shows the UV–vis absorption spectra of the BENDA–ctDNA complex from 0 h to 12 h measured with a 2 h interval, along with a detailed analysis of the absorbance at 232 nm for each spectral line. As seen in the figure, the black spectral line (reaction time 5 min, noted as initial 0 h) was considered as the control spectrum, and after sequential addition of ctDNA solution, a hypochromic effect first occurred in the BENDA–ctDNA complex, i.e., the absorbance values of the spectra measured from 0 h to 4 h were all lower than the control spectra; afterward, a hyperchromic effect occurred, i.e., the absorbance values of the spectra from 6 h to 12 h were all higher than the control spectra. Figure 8d shows the trend of the absorbance values of the BENDA–ctDNA complex from 0 h to 12 h. A reasonable explanation for this phenomenon may be that BENDA molecules are first attracted to the phosphate backbone of ctDNA by electrostatic forces, and electrostatic binding to it leads to a hypochromic effect in the spectrum, while a part of BENDA, once saturated at the electrostatic site, may interact with the bases in ctDNA, leading to a spectrum showing a hyperchromic effect [42].

### 2.4. Molecular Docking

**Figure 9:**
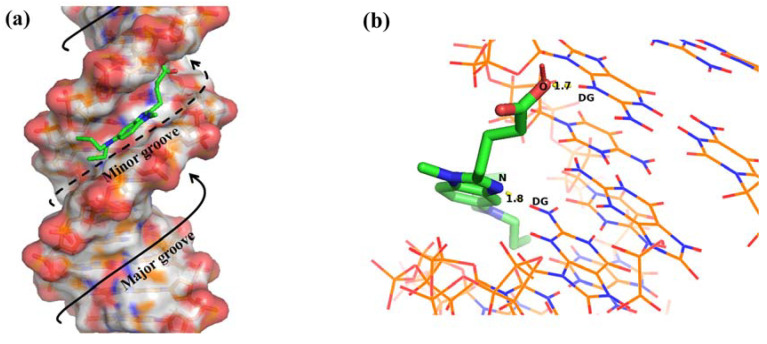
(**a**) Binding of BENDA (green) in the DNA minor groove obtained by molecular docking simulation; (**b**) site of hydrogen bond formation, DG: Deoxyguanosine.

The simulated binding conformations between BENDA and DNA finally calculated in AutoDock software have 29 cluster class combinations, each containing 1 to 5 conformations. As shown in Figure 9, we selected the docking conformation with the lowest binding energy for analysis and visualized the binding simulations using Pymol to visualize the calculated results between BENDA and DNA, which can also be used to verify the results obtained from spectroscopic studies [43]. Molecular docking was used to predict potential binding interactions between BENDA and DNA at the molecular level, while SERS spectroscopy allowed us to experimentally probe the vibrational modes of the drug and DNA upon interaction. Several factors might contribute to this discrepancy between molecular docking predictions and SERS experimental observations. Despite these discrepancies, the molecular docking results are presented as a basis for the proposed mechanism due to their ability to provide valuable insights into potential binding interactions at the molecular level. While molecular docking is a powerful tool for predicting binding modes and interactions, we emphasize the need for caution when directly interpreting these results as experimental evidence. In conclusion, the combination of molecular docking and SERS spectroscopy provides a comprehensive approach to studying drug–DNA interactions. The differences between the results obtained from both approaches highlight the complexities of the system under investigation.

The Lamarckian genetic algorithm yields a minimum binding energy of −7.22 kJ/mol for the interaction between the two, with the intermolecular docking region shown in Figure 9a, which clearly shows that the small BENDA molecule binds to DNA in the small groove region, especially in the GC base-rich region. Figure 9b further elucidates the hydrogen bonding sites and binding lengths between the base pairs in the ligand BENDA and the recipient DNA, i.e., the oxygen atom (O) of the hydroxyl group (-OH) in BENDA and the nitrogen atom (N) in the imidazole ring form hydrogen bonding interactions with the imino (-NH-) and amino (-NH_2_) in the guanine base, forming hydrogen bonds with lengths of 1.7 Å and 1.8 Å, respectively. The results indicate that BENDA binds to DNA in a groove mode and hydrogen bonding plays an important role in the binding process of BENDA to DNA.

### 2.5. Mechanism of Action of BENDA and DNA

**Figure 10:**
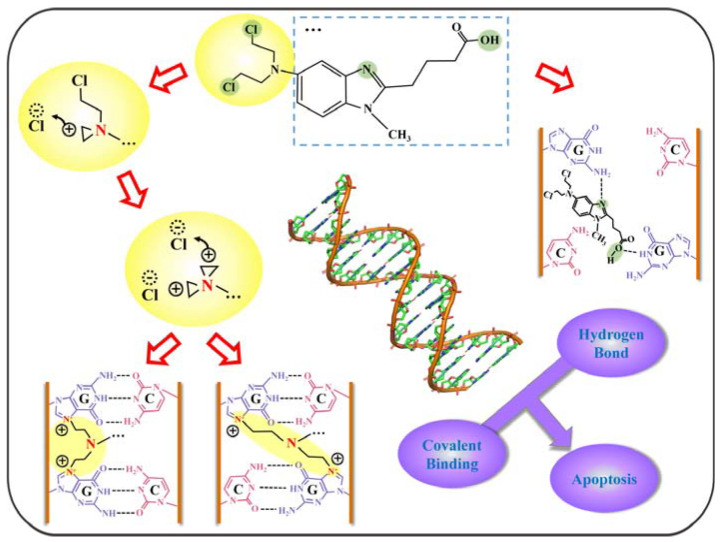
Diagram of the active sites of BENDA and binding modes to DNA molecule.

Based on the above experimental results, a model of the mechanism of action of BENDA with DNA can be deduced. Since the amount of information observed at the A and T bases is minimal and the C base is not a major site of action, we will only discuss information on G base interactions. As shown in Figure 10, BENDA possesses four active sites (at the light green circles), namely the two Cl atoms in the alkylation group of 2-chloroethylamine, the N atom on the benzimidazole ring, and the O atom of the hydroxyl group (-OH) on the side chain of butyric acid. In this case, the process with the alkylation group of the G base is shown in the route on the left side of Figure 10, where the Cl atoms in the BENDA molecule detach one after another, making the detached site extremely electrophilic and thus covalently bound to the N7 site of the G base. The route on the right side of Figure 10 shows the location of the hydrogen bonds formed between the N atom in the benzimidazole ring and the O atom in butyric acid (at the light-yellow rectangular box), which create hydrogen bonds with the amino (NH_2_) and imino (NH) groups in the G base, respectively, enhancing the drug–DNA interaction even more. These two different DNA action pathways together lead to intra- and interstrand cross-linking of its double helix, which ultimately triggers the apoptotic mechanism of DNA. Notably, covalent interaction (i.e., alkylation binding)-induced inter- and intra-strand cross-links play a dominant role, while non-covalent interaction (groove binding and electrostatic binding) plays a secondary role.

## 3. Materials and Methods

BENDA was used in its hydrochloride form and its purity was >98% as determined by 1H-NMR and HPLC; it was purchased from Hanxiang Biotechnology Co., Shanghai, China. The original solution (concentration 0.1 mol/L) is prepared in the compound Dimethyl Sulfoxide (DMSO, CAS: 67-68-5) with a concentration of 0.25% and fully dissolved using an ultrasonic machine, after which it is stored at room temperature (generally 25 °C) away from light. DMSO was purchased from MP Biomedicals, Irvine, CA, USA. ctDNA (CAS: 73049-39-5) was purchased from Sigma-Aldrich (St. Louis, MO, USA) and prepared as a stock solution at a concentration of 0.1 mol/L (concentration determined by the molar absorption coefficient at 260 nm [44]) and stored at 4 °C in a refrigerator protected from light. Absorption coefficient at 260/280 nm (A260/280) and purification of ctDNA degree values were both greater than 1.8, indicating that the prepared samples were protein-free and the solutions were pure [45]. ctDNA solution of corresponding concentration can be prepared according to the experimental requirements, and the method of determining the concentration before and after dilution is the same. Oxalic acid reagent was obtained from Xi’an Sanpu Chemical Reagent Co., Xi’an, China. Except for the substrate preparation process, the whole experimental procedure was carried out at room temperature, and fresh ultrapure water was used for the preparation of solutions. Origin software (Origin 2017. OriginLab Corporation. Northampton, MA, USA) was mainly used for data processing and analysis.

### 3.1. SERS

The above AuNPs/ZnCl_2_/NpAA substrates were used as SERS substrates and a laser confocal Raman system (Alpha 500R, WITec, Ulm, Germany) was used as the SERS spectral acquisition system for the specific study of the interaction between BENDA and DNA. The Raman spectra of the solids, i.e., BENDA powder and DNA fibers, were acquired by a laser at 532 nm, and the solution spectra acquisition was replaced by a laser at 785 nm, with both objectives at 20× and a grating at 600 g/mm, with a set exposure time of 5 s and 2 exposures. The system can achieve spectral resolution of ~3 cm^−1^ and spectral repetition rate of ~0.02 cm^−1^. The collected experimental data were preprocessed using WITec Project Four software, and the final spectral results were plotted by Origin software.

In order to keep the same conditions for the experiments, the substrates of the measured substances (BENDA, ctDNA, and BENDA–ctDNA) were all cut from the same negative sheet. For the detection of BENDA and ctDNA, the cut sheets of substrate were immersed in the solution to be measured for 1 h, while the BENDA–ctDNA complexes with different molar ratios needed to be incubated for 5 h. Then, the substrates were dried at room temperature and ready for SERS spectroscopy after complete adsorption of the molecules. Each set of spectra was obtained by averaging 10 valid data.

### 3.2. Scanning Electron Microscope

The NpAA and ZnCl_2_/NpAA solid substrates were characterized morphologically using a Japan Hitachi-su-8010, CRESTEC-CABL-9000C field emission scanning electron microscope (Thermo Fisher Scientific, Waltham, MA, USA), respectively, and the modified AuNPs/ZnCl_2_/NpAA substrates were analyzed for elemental species using the Energy Dispersive X-Ray Spectroscopy (EDX) energy spectrometer included in the SEM system.

### 3.3. UV–Vis Absorption Spectroscopy

We collected the UV–vis Absorption Spectroscopy of the drugs using a DS-11 UV-vis spectrophotometer. ctDNA concentration and reaction time were used as factors affecting the spectra of the samples. The data were taken three times for each set of experiments, and the average value was taken for the study and analysis.

- ctDNA concentration

In the first step, a BENDA solution with a concentration of 1 × 10^−5^ mol/L was prepared, and 1 mL of this solution was added to the centrifuge tube using a pipette. In the second step, replace the small-range pipette to add 10 μL of ctDNA solution (concentration of 1 × 10^−2^ mol/L) to the centrifuge tube in batches, so that the concentrations of ctDNA solution in the mixed system are in order: 0.0, 1.0 × 10^−4^, 2.0 × 10^−4^, 3.0 × 10^−4^, 4.0 × 10^−4^, and 5.0 × 10^−4^ mol/L. Each time, after the dropwise addition of ctDNA solution, the centrifuge tube needed to be kept in an oscillator for 10 s, and then it was left to stand for 15 min to wait for the full reaction of the drug. Finally, the UV–vis absorption spectra of each group of mixed solutions were measured sequentially in the wavelength range of 220 nm to 400 nm.

- reaction time

In the first step, 50 μL of each BENDA solution and ctDNA solution (both at a concentration of 1 × 10^−4^ mol/L) were added to the same well of a 96-well plate. In the second step, the well plates were sealed from light and placed on a shaker for 5 min, where the moment after 5 min was recorded as 0 h. Finally, the UV–vis absorption spectra of the BENDA–ctDNA mixture solution were collected sequentially between 0 h and 12 h with an interval of 2 h to study the mechanism of the interaction between the two with time.

### 3.4. Molecular Docking

Molecular docking experiments of BENDA and DNA were implemented by AutoDock software (AutoDock 4.2.6. Scripps Research Institute, San Diego, CA, USA), which has several algorithms to calculate information such as binding conformation and force between the two substances. The crystal structure of the receptor (i.e., DNA molecule) was collected from the Protein Structure Data Bank (https://www.rcsb.org accessed on 30 August 2023) for a B-type DNA fragment (PDB ID: 425D) with sequence d(ACCGGTACCGGT)_2_, the closest conformation to DNA in the cell. The 3D structure of the ligand (BENDA, Compound CID: 65628) was collected from the PubChem database (https://pubchem.ncbi.nlm.nih.gov/ accessed on 30 August 2023). The selected grid point position is 112 × 60 × 60 (that is, x × y × z), and the grid point spacing is 0.375 Å. The macromolecular DNA was set to remain rigid during docking and random docking calculations were performed for the receptor and ligand using the Lamarckian genetic algorithm, which was run 50 times. The docking conformation with the lowest binding energy (i.e., most probable) was selected among the final binding cluster classes, and the docking results were visualized and analyzed using PyMol 2.5.2 software.

## 4. Conclusions

In this study, we used SERS spectroscopy, UV–vis absorption spectroscopy, and molecular docking to investigate the binding mechanism of BENDA interacting with DNA. SERS experimental results showed that under low molar ratio, the positions of two Raman peaks 802 cm^−1^ (representing deoxyribose) and 1069 cm^−1^ (representing phosphate main chain) changed, which together confirmed the existence of some external binding between BENDA and the sugar–phosphate main chain in the DNA structure. The intensity of the peak position of the groove marking band (1494 cm^−1^) in the spectrum gradually decreased and red shifted to 1485 cm^−1^, indicating that the hydrogen bonding environment of the G base had changed, and it was presumed that the N7 atom of the G base was the action site of BENDA when binding with DNA. As the molar ratio increased, the changes in peak intensity at 1185, 1250, 1342, 1378, and 1431 cm^−1^ confirmed the structural polycondensation of DNA in the BENDA solution, and the gravitational attraction between the double helix chains increased and decreased under high concentration conditions. The results of UV–vis spectroscopy showed that the interaction between BENDA and ctDNA was an external bond, that is, both groove binding and electrostatic binding. The molecular docking results showed that BENDA was bound in the small groove region of DNA, and the N and O atoms in the structure formed intermolecular hydrogen bonds with the base G. Finally, we elucidate that there are four active sites in the structure of BENDA, which are two Cl atoms in the alkylation group, N atom on the benzimidazole ring, and O atom on the side chain hydroxyl (-OH) of butyric acid. The alkylation group is covalently bound to DNA via N7 on the G base. The latter two assisted in strengthening intra- and inter-chain crosslinking through hydrogen bonding force, and together they induced eventual apoptosis.

## Figures and Tables

**Table 1:** Assignment of Raman peaks of BENDA-ctDNA complexes (600–1700 cm^−1^).

BENDA-DNA/cm^−1^	Attributed Species	Assignment
802	DNA	Phosphate backbone
1069	DNA	C-O stretch of deoxyribose
1149	BENDA	HCC, CC, CO
1185	DNA	extra-base C-N stretching
1250	DNA	A, C
1342	DNA	A
1378	DNA	T, A, G
1431	DNA	A
1494	DNA	G, A
1563	BENDA	C-C, H-C-H

## Data Availability

Not applicable.
